# Genetic Evaluation of Heteroresistance among Carbapenem-Susceptible Clinical Isolates of Enterobacterales

**DOI:** 10.1155/2024/5014876

**Published:** 2024-08-26

**Authors:** İpek Koçer, Mehmet Erinmez, Yasemin Zer

**Affiliations:** ^1^ Department of Medical Microbiology SANKO University School of Medicine, Gaziantep 27090, Türkiye; ^2^ Department of Medical Microbiology Gaziantep University School of Medicine, Gaziantep 27310, Türkiye

## Abstract

Carbapenems currently serve as the last line of defense when treating serious infections caused by multidrug-resistant Enterobacterale*s* species; however, heteroresistance of these species is thought to cause failure in the treatment with these broad-spectrum antibiotics. This study was designed to determine the prevalence of carbapenem heteroresistance and associated genotypic modifications among phenotypically meropenem-susceptible *Escherichia coli* and *Klebsiella pneumoniae* isolates. A total of 204 isolates of *E. coli* (*n*: 118) and *K. pneumoniae* (*n*: 86) from various clinical samples were included in this prospective experimental study. Identification and antimicrobial susceptibility testing of the isolates were performed by VITEK® (bioMérieux, France). Strains that were found susceptible to carbapenem group antibiotics (meropenem, imipenem, and ertapenem) with automated system were further investigated by disk diffusion method. The isolates with discrete colony growth within the clear inhibition zone among phenotypically meropenem-susceptible strains were tested for heteroresistance with the “gold standard” population analysis profile-area under the curve (PAP-AUC) method. In addition, heteroresistant isolates were analyzed for the presence of carbapenemase genes with in-house PCR method. The heteroresistance prevalence rate was 3.5% for *E. coli* and 18.1% for *K. pneumoniae*. The presence of heteroresistance in a total of 10 meropenem-susceptible isolates (*E. coli*, *n*: 4; *K. pneumoniae*, *n*: 6) was confirmed by the PAP-AUC method. The most frequently detected carbapenemase in heteroresistant isolates was OXA-48 (6/10), followed by NDM-1 (2/10). Meropenem is frequently preferred as initial empirical monotherapy in most of Gram-negative infections in adult and pediatric patients. The presence of heteroresistance against meropenem is too important to ignore, and for this reason, it seems beneficial to prefer combined treatment regimens in clinical practice.

## 1. Introduction

Today, antimicrobial resistance poses a serious risk to public health [1]. When treating severe infections caused by multidrug-resistant Enterobacterales species, carbapenems are the last line of defense among current antibiotics [2]. Resistance rates have increased as a result of the extensive use of carbapenem antibiotics and carbapenem-resistant Enterobacterales (CRE) were added to the list of bacteria that require novel antibiotics urgently by the World Health Organization (WHO) in 2017 [3].

Another issue regarding Gram-negative bacteria, like *K. pneumoniae*, *E. coli*, and *Acinetobacter* spp. is the concept of heteroresistance [4]. Heteroresistance is a concept that indicates different responses to antibiotics within a bacterial community and is defined as the population-wide variation in antibiotic resistance [5]. Although the exact mechanisms of heteroresistance are not known and they remain uncertain in international standards such as The European Committee on Antimicrobial Susceptibility Testing (EUCAST), its reflection in the clinic is thought to cause treatment failure [6]. For instance, even though the isolates of *E. coli* and *K. pneumoniae* were found to be carbapenem susceptible, distinct colonies that grow in the inhibitory zone are considered to be heteroresistant [7]. Although definitive detection methods have not been approved for heteroresistance; the gold standard in the determination of heteroresistance is the population analysis profile-area under the curve (PAP-AUC) method [5, 8]. Showing the relationship between heteroresistance and clinical prognosis in terms of both the spread of resistance and treatment success will reveal the importance of heteroresistance. This study aimed to investigate the heteroresistance profile of meropenem-susceptible *E. coli* and *K. pneumoniae* isolates by genotype analysis and the PAP-AUC method.

## 2. Materials and Methods

This study was carried out at a third-step university hospital in Gaziantep, Turkey, between March 2022 and March 2023 and was approved by the Clinical Research Ethics Committee (Date: 23.02.2022 and Decision No: 2022/70). A total of 204 *E. coli* (*n*: 118) and *K. pneumoniae* (*n*: 86) strains isolated from various clinical samples were included in the study. *E. coli* ATCC 25922 strain was used for quality control. The Mann–Whitney *U* test was used for statistical analysis and a *P* value less than 0.05 is deemed to be statistically significant.

### 2.1. Identification and Antimicrobial Susceptibility Testing

The automated VITEK® (bioMérieux, France) system was used for the identification and antimicrobial susceptibility of the isolates. Strains which were susceptible to meropenem with an automated system were further investigated with the Kirby–Bauer disc diffusion method as follows: the bacterial suspension prepared at 0.5 McFarland standard was spread onto Mueller Hinton agar (MHA; Oxoid, USA) with the help of a sterile swab and antibiotic discs containing 10 *μ*g meropenem (Oxoid, USA) were placed on the surface of the medium. After 24 hours of incubation at 35 ± 2°C, the diameters of the inhibition zones were measured and evaluated in accordance with EUCAST guidelines [9]. Bacterial colonies detected within the inhibition zone of isolates which were sensitive to meropenem in the disc diffusion test were further examined for the presence of heteroresistance. Patient files underwent an ambidirectional assessment to show the time-dependent variation of antibiotic susceptibility results of the patients with heteroresistant colony formation.

### 2.2. Analysis of Carbapenem Heteroresistance: PAP Curves and Difference between MICs

Based on Clinical and Laboratory Standards Institute (CLSI) criteria, suspicious colonies for the presence of carbapenem heteroresistance detected by the Kirby–Bauer disk diffusion method were stocked in the skim-milk medium at −20°C [10]. Population profile analysis method (PAP) was used for the determination of heteroresistance as previously described [8]. A meropenem-susceptible isolate, *E. coli* ATCC 25922 (MIC: 0.016 *μ*g/mL) quality control strain, and a meropenem-resistant *E. coli* (MIC: 16 *μ*g/mL) strain from the clinical strains were employed as control strains in the PAP investigation. Bacterial suspensions of the isolates were prepared in Mueller Hinton broth (MHB) at 0.5 McFarland turbidity standard. Then, suspensions containing 10^7^, 10^6^, 10^5^, 10^4^, 10^3^, and 10^2^ CFU/mL bacteria were obtained with 10-fold serial dilutions. The prepared bacterial suspensions were inoculated onto MHA media containing increasing concentrations of meropenem (i.e., 0.125, 0.25, 0.5, 1, 2, 4, 8, 16, 32, 64, 128, and 256 *μ*g/mL). Additionally, to compare control and colony numbers, suspensions containing 10^3^ and 10^2^ CFU/mL bacteria were prepared and inoculated into antibiotic-free MHA medium. After an incubation of 48 hours at 35 ± 2°C, bacterial colonies grown on each culture plate were counted. A graph was created according to colony numbers and antibiotic concentrations (0–256 *μ*g/mL); the logarithms of the colony numbers on the *y*-axis and the antibiotic concentrations on the *x*-axis, and a curve were obtained for each strain. The curves of resistant and susceptible isolates from the studied isolates were shown in a graph to resemble resistant and susceptible isolates. PAP-AUC analysis was performed [11]. Since there are no PAP/AUC ratios determined for carbapenem heteroresistance in Enterobacterales species, if the area under the curve in the graph was between carbapenem-susceptible and resistant isolates, the isolate was accepted heteroresistant.

Furthermore, we applied an interpretation criterion based on standard antibiotic sensitivity testing to validate heteroresistance. El-Halfawy et al. proposed that >8-fold difference between the lowest concentration exhibiting maximum inhibition and the highest noninhibitory concentration may be regarded as heteroresistance [5]. Broth microdilution results of the clinical strains and discrete colonies within the inhibition zones were compared.

### 2.3. Determination of Carbapenem Resistance Genes

**Figure 1:**
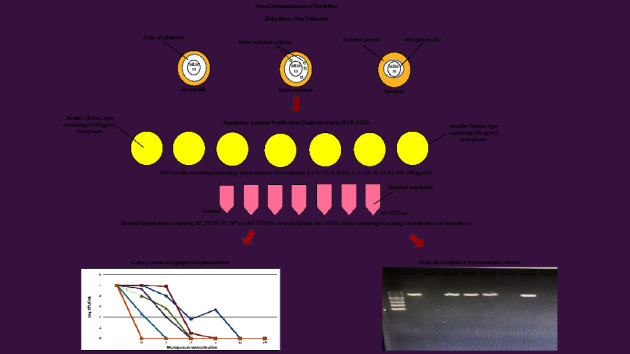
Graphic schematization of general workflow.

The isolates, which were detected as suspicious for the presence of carbapenem heteroresistance by the Kirby–Bauer disk diffusion method, were evaluated by in-house polymerase chain reaction (PCR) method for the presence of *blaKPC*, *blaNDM*, *blaOXA48*, *blaVIM*, and *blaIMP* carbapenemase genes as previously described [12]. To prepare a bacterial suspension, 10X Taqbuffer (Thermo Scientific, USA) diluted 1/10 with distilled water was used. Three to four colonies of each isolate were transferred along with 250 *µ*L of diluted buffer to create a bacterial suspension at 1 McFarland turbidity standard into 1.5 mL Eppendorf tubes. The total amount of the reaction mixture per isolate was 20 *µ*L; 10 *µ*L of 2X PCR master mix (Thermo Scientific, USA), 1 *µ*L of target precursor primer, 1 *µ*L of target reverse primer, 1 *µ*L of bacterial suspension, and 7 *µ*L of PCR grade water. In the thermal cycler, the first cycle consisted of 5 rounds of 3 minutes at 95°C, 15 seconds at 95°C, 30 seconds at 52°C, and one minute at 72°C, and the second cycle consisted of 20 rounds of 15 seconds at 95°C, one minute at 50°C, and one minute at 72°C. General workflow was demonstrated in Figure 1.

## 3. Results

The distribution of 204 Enterobacterales isolates (*E. coli*, *n*: 118; *K. pneumoniae*, *n*: 86) according to sample type was as follows: 130 (63.7%) urine, 23 (11.2%) tracheal aspirate, 22 (10.7%) wound, 17 (8.3%) blood, and 12 (6.1%) other in the study.

### 3.1. Kirby–Bauer Disk Diffusion Test Results

**Figure 2:**
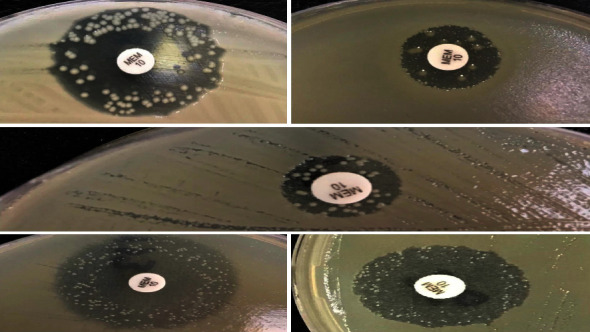
Representative images of Kirby–Bauer disk diffusion tests.

By Kirby–Bauer disk diffusion method, 146 (71.6%) of the isolates were found to be susceptible to meropenem. Ten (6.8%; *E. coli*, *n*: 4; *K. pneumoniae*, *n*: 6) of the meropenem-susceptible isolates showed colony formation within the inhibition zone and these were further evaluated for the presence of carbapenem heteroresistance. Also, the representative images of Kirby–Bauer disk diffusion tests are shown in Figure 2.

### 3.2. PAP Results and MIC Comparisons

**Figure 3:**
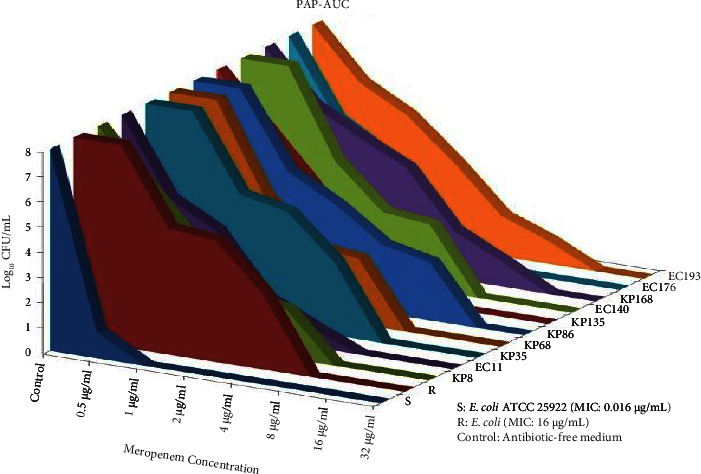
Graphical presentation of population profile analysis and areas under the curve (PAP-AUC). S: susceptible; R: resistant; KP: *K. pneumoniae*; EC: *E. coli*.

Colony counts of all isolates investigated for the presence of carbapenem heteroresistance at different meropenem and bacterial concentrations were plotted and shown in Figure 3. In addition, when the areas under the curve were compared in the graph, the areas under the curve of all isolates that were considered suspicious for heteroresistance by the disc diffusion method were determined as >*S* and <*R*, and the isolates were considered carbapenem heteroresistant. Also, there was *a* ≥ 8-fold difference between the MICs of the original strains and discrete colonies within the inhibition zones (Table 1). The prevalence of carbapenem heteroresistance among the 146 meropenem-susceptible isolates included in our study was 6.8% in general (3.5% for *E. coli* (*n*: 4 among 113 meropenem-susceptible isolates), 18.1% for *K. pneumoniae* (*n*: 6 among meropenem-susceptible 33 isolates)).

### 3.3. Presence of Carbapenem Resistance Genes

**Figure 4:**
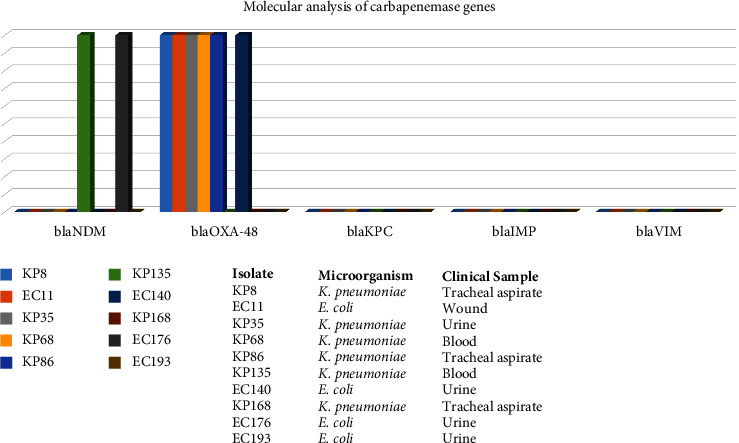
Molecular analysis of carbapenemase genes.

According to PCR results, the *blaOXA-48* gene was found in 6 (60%) and the *blaNDM* gene was found in 2 (20%) of 10 isolates detected as heteroresistant to meropenem. No resistant gene was detected in the remaining 2 isolates (Figure 4).

### 3.4. Evaluation of Clinical Findings

When the files of 10 patients who were found to be heteroresistant to carbapenem were evaluated retrospectively, it was determined that all patients had received meropenem treatment previously, because of that carbapenem is frequently the preferred initial antibacterial therapy protocol in our hospital. While receiving meropenem therapy, resistance to meropenem emerged in one patient with recurrent *K. pneumoniae* growths in his tracheal aspirate samples. Although no resistance development to meropenem was observed in 3 patients while receiving meropenem treatment, recurrent growth was observed despite the treatment. In the remaining 4 patients, no development of resistance or recurrent growth was observed during meropenem treatment.

## 4. Discussion

Antibiotic resistance rates among Gram-negative pathogens are increasing in hospital-acquired and community-associated infections. As a result, mortality rates due to resistant pathogens are gradually increasing all over the world and are competing with chronic diseases. According to the Centers for Disease Control and Prevention (CDC) 2019 report, resistant pathogens cause more than 2.8 million infections in the USA; the death toll was reported to be more than 35,000 [13]. Although carbapenems are the most effective drugs for extended-spectrum beta-lactamase- (ESBL-) producing *E. coli* and *K. pneumoniae* isolates, resistance develops against these agents due to their frequent use in treatment. It has been reported that carbapenem resistance is more common in *K. pneumoniae* isolates than *E. coli* in hospital-associated infections in Europe and Turkey, and Turkey is considered “endemic” in terms of CRE [14, 15]. Carbapenemase production is the most important mechanism in carbapenem resistance, and expression of KPC, IMP, VIM, NDM, and class *D* beta-lactamases (OXA-48 and its variants) occurs frequently [16]. According to recent studies, OXA-48 is still the most common carbapenemase enzyme producer, and it is noted that there is an increase in NDM-1 producers [17, 18]. Consistent with previous data, the most frequently detected carbapenemase enzyme in our study was OXA-48, followed by NDM-1.

It has been suggested that heteroresistance plays a significant part in carbapenem resistance since it is one of the factors underlying treatment failures [7]. While carbapenem resistance in Enterobacterales is frequently encountered in the literature, there are limited studies on carbapenem heteroresistance. Consistent with the data of our study, Sun et al. determined the frequency of meropenem heteroresistance in invasive *E. coli* isolates as 3.9% and drew attention to the importance of early detection of ESBL production as a risk factor for heteroresistance [19]. In carbapenemase-producing *K. pneumoniae* isolates which detected meropenem-susceptible by phenotypic methods, heteroresistant subpopulations that can lead to treatment failure with meropenem alone have been found to significantly increase the expression of the *blaKPC* gene [20]. In another study conducted on VIM-producing *K. pneumoniae* isolates; carbapenem susceptibility results with phenotypic methods gave different results and this was due to heteroresistant subpopulations [21]. Zavascki et al. pointed out that the *blaNDM-1* producer strains can be detected susceptible to carbapenems by phenotypic methods and that the detection of heteroresistant subpopulations by PAP method is important in terms of infection control [22]. *blaKPC-2* and *bla-OXA-48* genes were found to be the cause of carbapenem heteroresistance by Sancak et al. when whole genome analysis was carried out on *K. pneumoniae* and *E. coli* isolates [23]. Lopez-Camacho et al. showed that meropenem heteroresistant OXA-48 producer *K. pneumoniae* isolates had mutations in the *OmpK36* porin gene [24]. In another study performed on carbapenemase-producing producing *K. pneumoniae* isolates, they were found to be phenotypically susceptible to carbapenems, but with the PAP method, they had heteroresistant subpopulations with mutations in the *OmpK36* porin gene [25]. In our study, 8 of the carbapenem-heteroresistant isolates were carrying carbapenemase-encoding genes (bla-OXA-48 and bla-NDM), while no carbapenemase gene could be detected in 2 of the isolates. It was suggested that these 2 isolates might express genes that cause modifications of outer membrane porins instead of carbapenemases.

The increase in the prevalence of carbapenem resistance despite infection control measures suggests that heteroresistant populations should be examined as a risk factor for carbapenem resistance [7]. The concept of heteroresistance refers to the presence of a resistant subpopulation of an isolate that is considered susceptible [5]. It is thought that the selection of more resistant subpopulations during antibiotic treatment may cause clinical problems. The lack of standard methods for the detection of heteroresistance and difficulties such as costs and workload suggests that current effort is insufficient to demonstrate the presence of these isolates [7]. According to our study, Enterobacterales isolates that exhibit a clear meropenem susceptibility in routine tests may harbor some meropenem-resistant subpopulations, which can be selected via carbapenem treatment. The heteroresistant subpopulation may be an overlooked resistance phenotype in routine susceptibility tests in clinical isolates [26]. Determining the precise numbers of these heteroresistant subpopulations through screening methods would be extremely crucial. At the same time, the interpretation of these isolates as susceptible in standard antibiotic susceptibility tests causes the resistance to become dominant after a while [5]. It is noteworthy that a higher rate of heteroresistance was encountered in *K. pneumoniae* compared to *E. coli* isolates in our study. In addition, examining the inconsistent results between the automated system and disc diffusion method, especially in heteroresistant isolates, is important for the correlation of *in vitro* susceptibility results and treatment success.

## 5. Conclusions

Because carbapenem-resistant Enterobacterales, which are mediated by carbapenemase enzymes, have been linked to worse clinical outcomes and greater in-hospital expenses in hospitalized patients, this study is critical to understanding the threat that these bacteria pose to public health. The trend for monitoring of heteroresistance to carbapenems among Enterobacterales increases the possibility of providing early warnings of carbapenem resistance, which may increase the success of treatment and shorten the duration of hospital stay in affected patients.

## Figures and Tables

**Table 1:** Broth microdilution results of clinical strains and discrete colonies within the inhibition zones.

Isolate	Microorganism	MIC value (*μ*g/mL)	MIC value of colony within inhibition zones (*μ*g/mL)	*p*
S	*E. coli ATCC 25922*	0.016	N/A	

R	*E. coli*	16	N/A	

KP8	*K. pneumoniae*	1	8	0.00012
EC11	*E. coli*	1	8	
KP35	*K. pneumoniae*	1	8	
KP68	*K. pneumoniae*	1	8	
KP86	*K. pneumoniae*	1	16	
KP135	*K. pneumoniae*	0.25	4	
EC140	*E. coli*	0.25	16	
KP168	*K. pneumoniae*	0.25	16	
EC176	*E. coli*	0.25	4	
EC193	*E. coli*	0.25	16	

N/A: not applicable.

## Data Availability

The data generated in the present study are included in the figures and/or tables of this article.
